# Monitoring physical health in child and adolescent mental health inpatient care: findings from the Y-Health longitudinal feasibility study

**DOI:** 10.3389/frcha.2026.1731769

**Published:** 2026-06-17

**Authors:** Rebekah Carney, Shermin Imran, Heather Law, Karina Lovell, Rathi Ravindrarajah, Clare Fenton, Nicola Green, Gillian Macafee, Olorunleke Erunkulu, Sophie Parker

**Affiliations:** 1 Youth Mental Health Research Unit, Greater Manchester Mental Health NHS Foundation Trust, Manchester, United Kingdom; 2 Division of Psychology and Mental Health, Faculty of Biology, Medicine & Health, University of Manchester, Manchester, United Kingdom; 3 Greater Manchester Mental Health NHS Foundation Trust, Manchester, United Kingdom; 4 Division of Nursing, Midwifery and Social Work, University of Manchester, Manchester, United Kingdom; 5 Leeds and York Partnership Trust, Leeds, United Kingdom; 6 Navigo Child and Adolescent Mental Health Service (Young Minds Matter), Northeast Lincolnshire, United Kingdom

**Keywords:** adolescent, health behaviour, longitudinal studies, mental health services, metabolic health, physical health

## Abstract

**Background:**

Young people with serious mental illness (SMI) face significant physical health inequalities, including increased risk of obesity, diabetes, and other preventable conditions, contributing to a 15–20-year reduction in life expectancy. Those in CAMHS inpatient care are particularly vulnerable due to high levels of psychological distress, psychotropic medication, obesogenic environments and high levels of adverse health behaviours. However, research in this setting is limited. This study aimed to assess the feasibility of monitoring physical health in CAMHS inpatient services, changes to physical and mental health over 6-months and explore young people's experiences of health care.

**Methods:**

A longitudinal feasibility study with a separate qualitative component was conducted across three CAMHS inpatient units, assessing physical and mental health at admission, 3-months and 6-months. Feasibility indicators and clinical outcomes including weight, cardiometabolic markers, lifestyle behaviours and wellbeing were collected through self-report, clinical records and physical health checks. A nested qualitative sub-study was conducted to explore participant experiences of physical health care in CAMHS.

**Results:**

Twenty-seven participants were recruited across three CAMHS inpatient units, with 93% (*n* = 25) retained at 6-month follow-up. Completion of assessments was high at baseline but declined post-discharge. At baseline 37% individuals were overweight or obese, which increased to 50% overweight or obese at follow-up. Metabolic conditions were frequently observed across all timepoints. Physical activity remained low, and diet quality was poor. Mental health and functioning appeared to improve, with reductions in HONOSCA scores suggesting improvements to overall health.

**Conclusion:**

This study provides the first longitudinal feasibility evidence on monitoring physical health among young people in CAMHS inpatient care, demonstrating high acceptability during admission and identifying substantial challenges with post-discharge monitoring. While descriptive trends indicated a high prevalence of physical health risk factors, the study was not powered to detect meaningful change. These findings highlight the need for integrated, developmentally appropriate models of physical health monitoring across inpatient and community services.

## Introduction

### Background and rationale

People with Serious Mental Illness (SMI)—including psychotic, schizoaffective and bipolar disorders—face significant physical health inequalities, leading to impaired functioning, reduced quality of life and a 15–20 year shortened life expectancy (1–4). This disparity comes from increased risks of non-communicable diseases (such as obesity or diabetes), adverse health behaviours, side effects of psychotropic medication, and poor physical healthcare often due to diagnostic overshadowing (1–5). This preventable mortality gap has been called an international “human rights scandal”, prompting action from the World Health Organization, National Health Service England, the Lancet Commission Group and CORE25PLUS5 which includes children and young people (3–8). Their recommendations focus on prevention, including better detection, monitoring and access to physical health interventions across inpatient and community settings.

Child and Adolescent Mental Health Services (CAMHS) support young people (typically up to age 18, sometimes 25) with emotional, psychological and mental health difficulties via community and specialist inpatient facilities. In the UK, CAMHS inpatient units provide short-term specialist care for young people with severe and complex mental health presentations, typically involving multidisciplinary treatment and psychotropic medication, with onward transition to community CAMHS, Child and Adult Mental Health Teams (CMHTs), or Early Intervention Services (EIS) following discharge. Individuals in inpatient care are at highest risk of poor physical health due to high levels of psychological distress and the use of obesogenic, sedative medication (e.g., antidepressants, antipsychotics, mood stabilisers) (9–11). Qualitative studies show that both young people and staff report barriers to healthy living in psychiatric care which are often “obesogenic”—characterised by limited movement, reduced exercise opportunities, restricted outdoor access and little control over diet (12–15). Our recent meta-analysis found that nearly half of CAMHS inpatients were overweight or obese, and showed early signs of metabolic risk (9). Routinely collected data shows rapid weight gain post-admission, which continues throughout the duration of admission (11, 15–17). High rates of adverse health behaviours such as physical inactivity and substance use are also common (9, 16, 17).

Despite early indicators of poor physical health, research in this area remains limited. Guidelines in secure CAMHS settings are often unclear, not child-informed, and staff frequently report confusion over responsibility for physical health (14). This contributes to diagnostic overshadowing and untreated/worsening conditions (10). Previous studies have had a small sample size, used only qualitative methods or had been based on inconsistent routine data, particularly when it comes to health behaviours such as physical activity, thus limiting generalisability and ability to draw meaningful conclusions. At the time of our study, no research had systematically or prospectively examined physical health trajectories post-admission.

### Aims and objectives

The primary aim was to conduct a longitudinal feasibility study to explore the physical health of young people admitted to CAMHS inpatient units over 6-months. The research questions and aims were:

1. To establish the feasibility of monitoring physical health of young people admitted to CAMHS inpatient units in the UK.
2. To assess the physical health and health behaviours of young people at the time of admission to CAMHS inpatient units.
3. To assess changes 3- and 6-months post-admission to CAMHS inpatient units.
4. To understand the experiences and beliefs about physical health care and monitoring in young people admitted to CAMHS inpatient units.

The present manuscript focuses on the feasibility and longitudinal quantitative outcomes (Aims 1–3); building on the baseline characteristics previously published [see Carney et al., 2025 (18)]. To enable a full account of the methods and results qualitative findings from Aim 4 are presented and reported separately.

## Methods

### Study design

Study processes were conducted and reported according to STROBE (Strengthening the Reporting of Observational Studies in Epidemiology) guidelines for reporting observational cohort studies [Von Elm et al., 2014 (19)]. A full reporting checklist can be found in the supplementary files. A longitudinal feasibility study was conducted over a six-month intake to CAMHS inpatient units, with outcomes collected at admission, 3-months, and 6-months post admission. The study included a separate qualitative sub-study to explore young people's experiences of physical health care (reported elsewhere). Three CAMHS inpatient units took part from; Greater Manchester Mental Health NHS Foundation Trust (GMMH NHS FT), Leeds York Partnership NHS Foundation Trust (LYPFT) and Humber Teaching NHS Foundation Trust. Funding came from the NIHR Applied Research Collaboration Greater Manchester (ARC-GM) as part of the mental health research stream and the study was a collaboration with the NIHR ARC Yorkshire and Humber (ARC-YH). Health Research Authority (HRA) approval (IRAS 288738) was granted by the Yorkshire and The Humber - South Yorkshire Research Ethics Committee [18/12/2020, Ref: 20/YH/0319]. Sponsorship was provided by the host site GMMH NHS FT. As this was a longitudinal, prospective study, clinical trial registration is not applicable and therefore a registration number is not specified. A detailed protocol and methodology are published elsewhere (18).

### Participants

Participants were recruited from CAMHS inpatient units between May 2021 and February 2022 (during the COVID-19 pandemic). We aimed to recruit consecutive admissions with the capacity to provide informed consent. While we could not control admission numbers, we sought to include as many eligible people as possible. Formal sample sizes were not set, as the study aimed to assess the feasibility of longitudinal analysis and describe clinical characteristics at admission, rather than test statistical significance. The inclusion and exclusion were as follows:

#### Inclusion criteria

**Figure 1:**
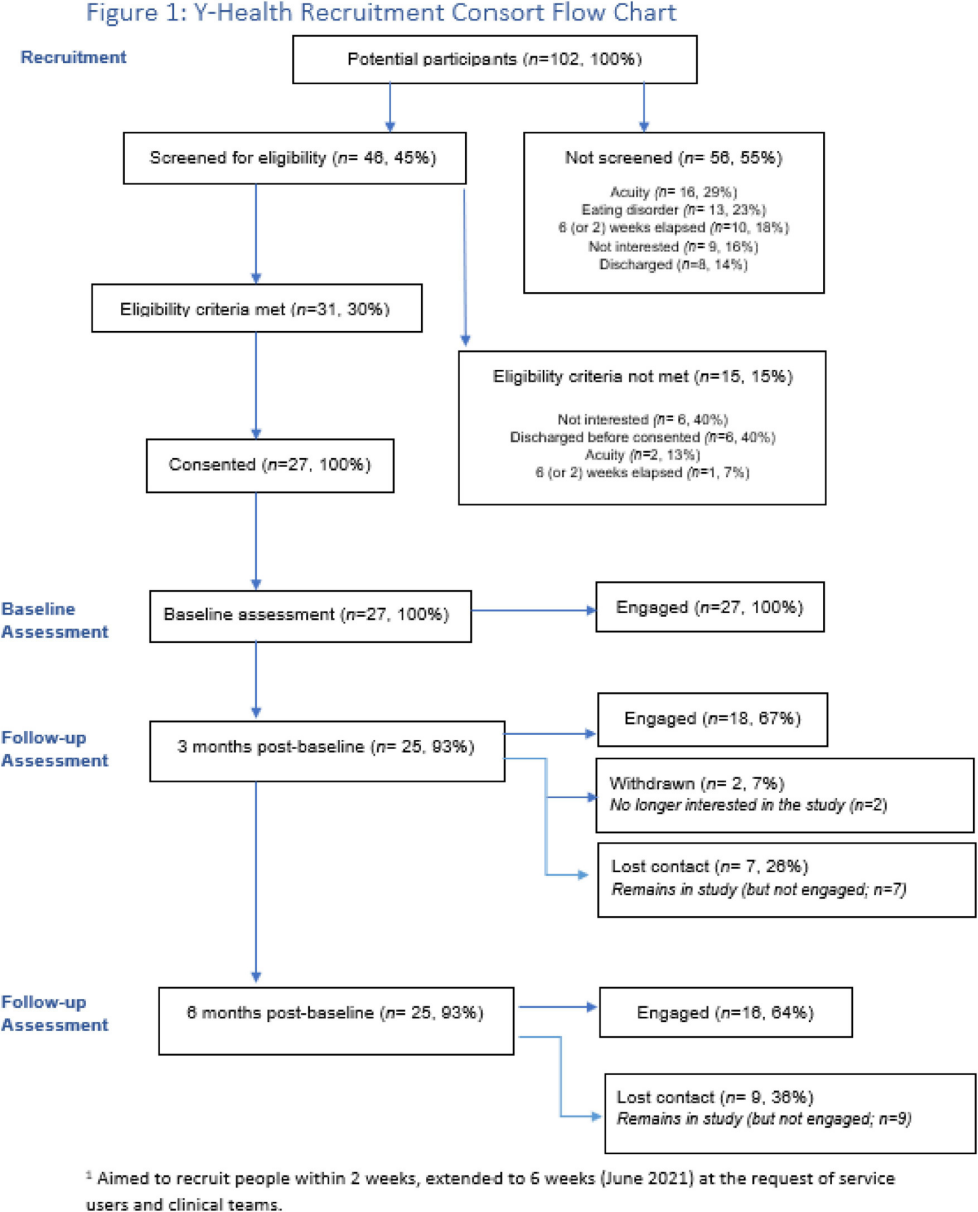
Y-Health recruitment consort flow chart.

- Currently receiving inpatient care from generic CAMHS inpatient units.
- Aged 14–18 years.
- Within 6-weeks of admission (within 2-weeks up until 04/06/2021).*(see footnote Figure 1)
- Capacity to provide informed consent.

#### Exclusion criteria

- Inability to provide informed consent (lack of capacity, cognitive impairment or severe communication difficulties).
- Severe eating disorder significantly compromising physical health.

To capture a diverse and inclusive sample, individuals who did not have sufficient command of English were eligible to take part using a translator.

Recruitment procedures are detailed in the protocol paper [Carney et al., under review (18)]. Briefly, researchers worked with clinical teams to identify eligible new admissions and obtain consent-to-contact. Study materials were available in shared areas for self-referral. Researchers provided study information to potential participants and obtained written informed consent prior to undertaking any research activities. Capacity to consent was assessed by clinical teams using Gillick Competency principles (Gillick v West Norfolk, 1985). Where necessary, parental consent and participant assent/consent were obtained together.

### Procedures

Assessments were conducted at three timepoints; baseline (within six weeks of admission), 3 months, and at 6-months. At each stage, participants completed sociodemographic, physical health and mental health assessments via self-report, researcher-administered questionnaires, or in-person physical health checks (see Table 1). Where in-person contact was not possible due to COVID-19 restrictions, assessments were conducted virtually or with support from clinical teams. Data collection was adapted where necessary to accommodate COVID-19 restrictions, which included the use of online data capture forms, support from clinical teams to obtain physical health data or perform brief assessments (such as blood pressure), or to facilitate appointments with participants. No changes were made to a participants' usual inpatient care, and no interventions were withheld. Participation was voluntary with the option to withdraw or decline assessments while remaining open to future follow-up.

**Table 1:** Y-Health SPIRIT diagram—schedule of events.

TIMEPOINT	Enrolment	Post allocation						
	-t_1_	1m	2m	3m	4m	5m	6m	6–12m
**ENROLMENT:**								
Eligibility screen	X							
Informed consent	X							
Baseline Assessments (measures listed below assessments)	X							
**FOLLOW UP:**								
3-month follow-up				X				
6-month follow-up							X	
**ASSESSMENTS:**								
Socioeconomic demographics	X			X			X	
Clinical demographics	X			X			X	
BMI (height/weight)	X			X			X	
Waist Circumference	X			X			X	
Blood Pressure	X			X			X	
Pulse	X			X			X	
Blood test	X			X			X	
ECG (Y/N)	X							
IFIS	X			X			X	
6-Minute Walk	X			X			X	
Simpaq	X			X			X	
Tobacco Use	X			X			X	
24-hour diet recall	X			X			X	
WHO ASSIST	X			X			X	
WHO-WI	X			X			X	
HONOSCA	X			X			X	
Qualitative Interview								X

Schedule of enrolment and assessments.

BMI, body max index; ECG, electrocardiogram; IFIS, International Fitness Scale; Simpaq, Simple Physical Activity Questionnaire; WHO-ASSIST, World Health Organization-The Alcohol, Smoking and Substance Involvement Screening Test; WHO-WI, World Health Organization Wellbeing Index; HONOSCA, Health of the Nation Outcome Scales for Children and Adolescents.

### Outcomes and assessments

The primary aim was to assess feasibility of the trial and associated processes including assessments and follow-up rates. Key markers of feasibility included recruitment rates, follow-up retention rates, completion of clinical outcomes and safety. The proposed primary outcome measure for a larger, statistically powered study was change in weight. Weight was selected due to its role as a modifiable metabolic risk indicator and predictor of long-term health outcomes. Psychotropic medications commonly prescribed in inpatient settings are obesogenic, with weight gain often occurring rapidly and preceding the onset of non-communicable diseases. Weight is also a simple, non-invasive measure, and one of the most frequently reported metabolic indicators, enabling comparisons across populations. Clinical outcomes included a purpose-built form to collect basic sociodemographic information (such as ethnicity, gender, education and living status) and clinically relevant information (such as mental health diagnoses, admission history, medication, physical health conditions), (See Table 1: SPIRIT Diagram). The following measures were collected.

#### Clinical demographics

- Mental health diagnoses were obtained from medical records (clinical teams were consulted directly if there was any uncertainty).
- Psychotropic medication, as defined by any drug prescribed for the treatment of mental health conditions which alter mood, perception and/or behaviour typically falling into one of the following categories: antidepressants, antipsychotics, anti-anxiety, hypnotics or mood stabilising drugs.
- Previous inpatient admissions to hospital for mental health conditions (duration and frequency).

#### Physical health assessments

- Body Mass Index (BMI; weight(kg)/height(m)_2_), waist circumference (cm), blood pressure, pulse rate.
- Blood Tests (random glucose/hba1c; lipids (High Density Lipoproteins – HDL, Low Density Lipoproteins – LDL, total cholesterol, triglycerides); serum prolactin levels; inflammatory markers (erythrocyte sedimentation rate – ESR), Full Blood Count (FBC).
- Electrocardiogram (ECG) and outcome or follow-on treatment or monitoring as recommended by a reviewing clinician or care team.
- International Fitness Scale [IFIS (20)], which is a validated self-report measure of perceived physical fitness in young people, showing moderate to good agreement with objective fitness indicators and acceptable reliability.
- 6-minute walk cardiometabolic fitness test.
- Comorbid physical health disorders or disabilities (self-reported or obtained via clinical notes).
- Concurrent medications and treatments for physical health conditions including any supplements prescribed.

#### Behavioural assessments

- The Simple Physical Activity Questionnaire [SIMPAQ (21)] to measure physical activity levels and sedentary behaviour. SIMPAQ is a brief, clinician-assisted tool developed specifically for use in mental health settings, demonstrating acceptable reliability and feasibility for assessing physical activity and sedentary behaviour.
- Smoking status (yes/no, current or lifetime use, quantity, type—including e-cigarettes but not vapes).
- 24-hour diet recall (22).
- WHO Alcohol, Smoking and Substance Involvement Screening Test [WHO ASSIST (23)].

#### Mental health outcomes

- WHO Wellbeing Index [WHO-WI (24)] is a brief well-validated measurement of subjective wellbeing with good internal consistency and sensitivity to change, and has been widely used in adolescent and clinical populations. It is tool used for children 9 years and over, consisting of statements relating to emotions and life satisfaction.
- Health of the Nation Outcome Scales for Children and Adolescents [HONOSCA (25, 26)] patient self-report, which is routinely used in CAMHS, reporting a range of behavioural, psychological, social, and functional outcomes. It has well established validity, inter-rater reliability and sensitivity to change across a range of clinical domains.

### Methods and data analysis

This feasibility study is not powered to detect statistically significant differences; therefore, our analysis is descriptive. The primary focus is on summaries of key indicators of success of the study (e.g., recruitment rates, retention, satisfaction and completion of outcome measures). Logistics data is reported according to STROBE (Strengthening the Reporting of Observational Studies in Epidemiology) guidelines for reporting observational cohort studies (19), including: the number of prospective participants, the number of people approached, subsequently deemed eligible and consented; the number completing baseline questionnaires; the number of participants assessed at follow-up (including any reasons for loss to follow-up); the number of participants providing “complete” clinical outcome data at each assessment. Analysis was conducted using STATA. Mean (s.d.) data was presented for continuous variables and N (%) for categorical variables. Descriptive summaries of demographics are reported, [full baseline characteristics can be found in Carney et al. (18)]. Descriptive data on change in outcomes between baseline, 3-months and 6-months are presented. Qualitative analysis was conducted using NVivo software [Version 13 (27)], using thematic analysis according to the methods outlined by Braun and Clarke (28). Data collection was completed in September 2022, baseline data and qualitative data are reported separately for full accountability and transparency.

## Role of the funding source

This work was supported by the National Institute for Health Research Applied Research Collaboration: Greater Manchester. The work was a collaboration with the National Institute for Health Research Applied Research Collaboration Yorkshire and Humber. The views expressed in this publication are those of the author(s) and not necessarily those of the National Institute for Health and Care Research for the Department of Health and Social Care.

## Results

### Participant flow

Between May 2021 and February 2022, 27 participants were recruited from a total of 102 CAMHS admissions across the units. The majority were recruited from GMMH NHS Foundation Trust (*n* = 19, 70% at baseline), followed by Humber Teaching NHS Foundation Trust (*n* = 5, 19%) and LYPFT (*n* = 3, 11%). Clinical teams assessed all admissions, 46 (45%) were screened in detail by the research team. The remaining 56 (55%) were not screened due to factors such as clinical acuity or inability to provide consent (*n* = 16), eating disorders (*n* = 13), elapsed time since admission (*n* = 10), lack of interest (*n* = 9), discharge prior to contact (*n* = 8). Of the 31 eligible individuals (67.4%), 27 (87%) consented to participate. Reasons for ineligibility at screening included early discharge to community or adult services before consent (*n* = 6), lack of interest on behalf of the young person (*n* = 6), acute illness (*n* = 2), or delay in contacting the individual (*n* = 1). Of the eligible participants, 27 (87%) provided informed consent and completed baseline assessments (See Figure 1). Recruitment was successful across all three NHS trusts.

### Baseline characteristics

At baseline, 70% (*n* = 19) of participants were female, 22% (*n* = 6) were male and 8% (*n* = 2) identified as other. Most participants were White (British or Other) (*n* = 18, 67%) and a smaller proportion identifying as mixed or other ethnic background. Most individuals were students (*n* = 20, 74%) and living with parents at the time of admission (*n* = 20, 74%). The average age upon admission was 16.1 years (±1.3) and it was the first admission for most young people (*n* = 21, 78%). Individuals who had previously been admitted to hospital had spent on average 33 days (±15) on inpatient units. Full baseline characteristics have been published elsewhere [Carney et al., in press (18)].

### Numbers analysed

Participants were successfully recruited from all three NHS foundation Trusts. At 3-month follow-up, 93% (*n* = 25) participants remained in the study, with 67% (*n* = 18) actively engaged. Two participants (7%) formally withdrew from the study stating that they were no longer interested in taking part as they had been discharged, and seven (26%) lost contact but remained enrolled in the study and available for contact. At 6-month follow-up, 93% (*n* = 25) participants were retained. Of these, 60% (*n* = 16) remained engaged, while 33% (*n* = 9) had lost contact. The primary reason affecting completion of assessments at follow-up was that participants were discharged from the inpatient unit and researchers were unable to make contact and arrange an appointment. Contact was attempted in multiple ways including phone calls, text messages, letters to home addresses, and attempts to contact via other individuals such as parents/caregivers, GPs, social workers. The study was not sufficiently powered to detect meaningfully significant differences, over time or between groups such as those receiving inpatient care compared with those discharged to the community.

#### Assessment completion

Table 2 presents the completion rate for assessments conducted at baseline (*n* = 27), 3-month follow up (*n* = 25) and 6-month follow up (*n* = 25), completion rates varied by measure type. Completion rates were consistently high at baseline (≥93%) for demographics, self-report measures and research assistant measures. Physical health measures were completed in full by 74%, and partially by 26%, with 100% completing at least the key physical health indicators (height, weight, BMI, and blood pressure).

**Table 2:** Completion of measures.

Assessment type	Measure	Baseline (*n* = 27)		3 months follow up (*n* = 25)		6 months follow up (*n* = 25)	
		n	%	n	%	n	%
Demographic Characteristics		25	93	24	96	20	80
Self-Report Measures		25	93	15	60	14	56
	Tobacco Use	25	93	18	72	15	60
	IFIS	25	93	17	68	14	56
	WHO-ASSIST	25	93	18	72	14	56
	WHO-WI	25	93	18	72	15	60
	HONOSCA	25	93	18	72	15	60
Research Assistant Administered Measures		25	93	14	56	10	40
	SIMPAQ	25	93	18	72	14	56
	24 Hour Diet Recall	25	93	18	72	14	56
	6-Minute Walk	5	19	1	4	1	4
Physical Health Measures: fully completed		20	74	6	24	3	12
Physical Health Measures: partially completed*		7	26	10	40	6	24
Physical Health Measures: any completed		27	100	16	64	9	36
	Height	27	100	15	60	19	76
	Weight	27	100	10	40	18	72
	BMI	27	100	10	40	18	72
	BMI Centile	27	100	10	28	18	72
	Waist circumference	5	19	2	8	5	20
	Blood pressure systolic	27	100	14	56	15	60
	Blood pressure diastolic	22	81	13	52	16	64
	Blood pulse rate	25	93	12	48	15	60
	HbA1c	19	70	6	24	13	52
	Prolactin	22	81	5	20	11	44
	HDL	21	78	6	24	11	44
	LDL	5	19	0	0	5	20
	Triglycerides	22	81	6	24	11	44
	Cholesterol	23	85	6	24	10	40
	ESR	0	0	0	0	0	0
	FBC	26	96	11	44	14	56
	ECG	27	100	8	32	7	28

*Partially completed includes any person who had completed one or more physical health measures.

At 3-months, completion rates decreased to self-report measures (60%), and researcher administered measures completed by 56%. Only 24% completed a full physical health assessment and 40% had partially completed a physical health check. At 6-months a similar pattern was observed with 56% of participants completing the self-report assessments and 40% completing researcher administered measures. Full physical health assessments were only completed in full by 12% of individuals and partially completed by 24% individuals—although completion of key physical health indicators had increased slightly compared with 3-month follow up. Blood-based measures (e.g., HbA1c, lipids, prolactin) and ECG were well captured at baseline (70%–100%) but showed reduced completion at follow-ups (20%–56%). Some measures were not well completed across all timepoints, for example waist circumference was missing or unavailable for most participants across all time points (completion at baseline *n* = 5, 19%; 3-months *n* = 2, 8%; 6-months *n* = 5, 20%).

Factors affecting completion of data included lack of contact with the participant following on from discharge, acuity or exacerbation of mental health difficulties, refusal from participant, or outcome not routinely collected/unavailable from the community mental health service.

### Outcomes

#### Clinical demographics

Full clinical demographics of participants have been presented and described in detail elsewhere [Carney et al., 2025 (18)]. See Table 3 for clinical characteristics. Primary diagnosis upon admission varied and consisted mainly of stress related disorders such as Post Traumatic Stress Disorder (*n* = 9, 33%), or mood disorders (*n* = 7, 26%). Some participants did not have a recorded mental health diagnosis, or they were in the process of receiving a diagnosis through assessment. One site used trauma informed care (TIC) formulation for all individuals (*n* = 4, 15%). TIC is a common approach often used in complex environments including secure psychiatric units and youth forensic teams which involves a multi-disciplinary team formulation to inform decision-making, care planning and treatment to understand a young person's past experiences of trauma and how it influences their behaviours and interactions with the environment and others. Sixteen individuals had more than one confirmed or suspected mental health diagnoses or identified as being neurodivergent. Additionally, 26% of individuals had a diagnosed neurodevelopmental disorder at baseline, rising to 36% at follow-up, see Supplementary Data. Patterns in diagnosis varied over time, and the amount of people with no formal diagnosis decreased at each follow-up timepoint, which was likely due to ongoing assessment and formulation. Additionally, the relatively low levels of young people diagnosed with psychotic disorders may have been due to the exclusion of individuals who were unable to provide informed consent. Fourteen individuals were prescribed antipsychotic medication and fourteen were prescribed antidepressants (52%), but these were not mutually exclusive. Most young people were prescribed more than one type of medication (*n* = 20, 74%), and this ranged from just 1 up to 5 different types of medication, including six individuals who were prescribed multiple antipsychotics (22%). Full range of psychotropic medication can be found in Supplementary Data.

**Table 3:** Clinical and demographic characteristics.

Participant demographics	Baseline *n* (%)/mean (s.d.)	3 month follow-up *n* (%)/mean (s.d.)	6 month follow-up *n* (%)/mean (s.d.)
Participants			
Total Participants	27 (100)	25 (100)	25 (100)
** **Greater Manchester Mental Health NHS Foundation Trust	19 (70)	18 (72)	18 (72)
** **Humber Teaching NHS Foundation Trust	5 (19)	4 (16)	4 (16)
** **Leeds and York Partnership NHS Foundation Trust	3 (11)	3 (12)	3 (12)
Gender			
** **Female	19 (70)	19 (76)	19 (76)
** **Male	6 (22)	5 (20)	5 (20)
** **Other/prefer not to say	2 (7)	1 (9)	1 (9)
Ethnicity			
** **White British	15 (56)	15 (60)	15 (60)
** **White and Black Caribbean	3 (11)	3 (12)	3 (12)
** **Other Mixed	3 (11)	2 (8)	2 (8)
** **Other White	3 (11)	2 (8)	2 (8)
** **Any Other Ethnic Group	3 (11)	3 (12)	3 (12)
Employment Status			
** **Student	20 (74)	16 (64)	17 (68)
** **Unemployed	3 (11)	3 (12)	3 (12)
** **Engaged in employment (part-time/voluntary)	2 (7)	2 (8)	2 (8)
** **Not Known	2 (7)	4 (16)	3 (12)
Living Status			
** **With Parents	20 (74)	15 (60)	16 (64)
** **Residential or Supported Accommodation	3 (11)	7 (28)	5 (20)
** **Foster Care	2 (7)	-	-
** **Other: Family or Lodging	2 (8)	3 (12)	2 (8)
** **Unclear or Unknown	-	-	2 (8)
Education Status			
** **GCSE or Equivalent	15 (56)	15 (60)	12 (48)
** **Not Known	4 (15)	4 (16)	5 (20)
** **Other: Working Toward GCSE	4 (15)	4 (16)	5 (20)
** **Other: College/Vocational	2 (7)	-	-
** **Further: A-level	2 (7)	2 (7)	3 (12)
Previous Admissions			
** **First Admission	21 (78)	17 (68)	17 (68)
** **1 or More Previous Admissions	6 (22)	7 (28)	7 (28)
** **Not known	-	1 (4)	1 (4)
Duration of Previous Admission (days)	32.9 (±15.3)	-	-
Destination Status			
** **Inpatient (Including Continued Stay or Readmission)	27 (100)	11 (41)	5 (19)
** **Remains inpatient	-	9 (33)	5 (19)
** **Inpatient Readmission^d^	-	2 (7)	-
** **CAMHS	-	9 (33)	8 (30)
** **CMHT/EIT	-	3 (11)	10 (37)
** **Day Service	-	2 (7)	-
** **Withdrawn	-	2 (7)	2 (7)
Diagnosis			
** **Mood Disorder	13 (48)	11 (44)	8 (32)
** **Disorders Associated with Stress	11 (41)	12 (48)	11 (44)
** **Neurodevelopmental Disorder	8 (26)	6 (24)	9 (36)
** **No Formal Diagnosis	4 (15)	2 (8)	2 (8)
** **Personality Disorder	2 (7)	4 (16)	4 (16)
** **Disruptive Behaviour or Dissocial Disorder	1 (4)	1 (4)	2 (8)
** **Other^a^	4 (15)	6 (24)	4 (16)
Medication			
** **Antipsychotics	14 (52)	12 (48)	12 (48)
** **Antidepressants	14 (52)	15 (60)	14 (56)
** **Antihistamines	14 (52)	10 (40)	2 (8)
** **Melatonin	6 (22)	8 (32)	7 (28)
** **Hypnotics/Anxiolytics	4 (15)	2 (8)	2 (8)
** **Stimulants	1 (4)	1 (4)	2 (8)
** **Vitamins	-	2 (8)	-
** **Sympathomimetics	-	1 (4)	1 (4)
** **Beta Blockers	-	-	1 (4)
Comorbid physical health diagnosis			
** **Respiratory Conditions (Asthma)	3 (11)	2 (8)	1 (4)
** **Metabolic Conditions^b^	12 (44)	10 (40)	11 (44)
** **Other^c^	4 (15)	5 (20)	7 (28)
** **Gastrointestinal/Genitourinary Conditions	2 (7)	3 (12)	2 (8)
** **Dermatological Conditions	2 (7)	-	2 (8)
** **Musculoskeletal Conditions	2 (7)	1 (4)	-
Number of Psychotropic Medications Prescribed			
** **Average	2.2 (±0.96)	2.4 (±0.99)	1.95 (±0.98)
** **1	7 (25.9%)	4 (16%)	5 (20%)
** **2	9 (33.3%)	11 (44%)	7 (28%)
** **3	9 (33.3%)	9 (36%)	8 (32%)
** **4	0 (-)	0 (-)	0 (-)
** **5	1 (3.7%)	1 (4%)	0 (-)

a Other diagnosis includes Disruptive behaviour or Dissocial disorder, Dissociative disorder, Psychotic disorder, Feeding and Eating disorder, and Sleep-Wake Disorders, Disorders due to Substance Abuse, Anxiety or Fear Related Disorders, Gender Incongruence, Obsessive Compulsive or Related Disorders, and Toxic or Drug Related Embryo Fetopathies.

b Metabolic Conditions (Anaemia, Cholesteatoma, Celiac Disease, Diabetes, Disordered Eating, Dyslipidaemia, Neutropenia, Impaired Renal Function, Vitamin D Insufficiency, Hyperthyroidism).

c Other physical health conditions include Gastrointestinal/Genitourinary Conditions (Constipation, Double Incontinence, Urinary Tract Infection, Phimosis, Rectal Bleeding) Dermatological Conditions (Psoriasis, Cholesteatoma, Infected Cyst, Ganglion, Corn), Musculoskeletal Conditions (Osgood-Schlatter Disease), Non-Epileptic Seizures, Haemochromatosis.

d Inpatient readmission refers to individuals who had been discharged to the community following the baseline assessment and subsequently readmitted to the unit.

All participants were receiving inpatient care at baseline. At 3-months, 41% (*n* = 11) remained in inpatient settings, including two individuals who had been discharged and subsequently readmitted. The remaining participants were discharged to community-based services including community CAMHS (*n* = 9, 33%), CMHT or Early Intervention Teams (*n* = 3, 11%), or were attending an inpatient day service (*n* = 2, 7%). At 6-months, most participants who were followed up had been discharged to community care, with only 19% (*n* = 5) remaining in inpatient settings. Community CAMHS continued to support 30% (*n* = 8) of participants and CMHT/EIT engagement increased to 37% (*n* = 10), likely due to increases in age.

#### Physical health outcomes

Table 4 reports physical health outcomes. At the time of admission, 37% of individuals were overweight, with 26% of these meeting the criteria for obesity (>95th percentile) based on BMI Centiles. Average weight increased from 64.5 kg to 69.3 kg at 6-month follow-up. BMI appeared to increase from 23.3 kg/m^2^ to 24.7 kg/m^2^ on average with a corresponding increase in BMI centiles (60.9 kg/m^2^–69.3 kg/m^2^). The proportion of participants classified as obese based on BMI centiles appeared to increase from 26% (*n* = 7) to 33% (*n* = 6). At both follow-up timepoints, half of the sample were overweight or obese and a high degree of variability was observed (e.g., 17–34, 6–99th centile). Descriptive data suggested increases in weight and BMI over time, with half of the sample classified as being overweight or obese at follow-up. Waist circumference data was limited across timepoints. Blood pressure remained stable across timepoints and remained within the normal range. Little fluctuations were observed in resting heart rate which again, was within normal range for this age group. Blood results varied across timepoints, with some decreases observed in HbA1c and haemoglobin, increases in prolactin and triglycerides, and little discernible changes in lipids or red/white blood cell count.

**Table 4:** Physical health measures.

Physical health measure	Baseline			3-month follow-up			6-month follow-up		
	N	N (%)/Mean (s.d.)	Range	N	N (%)/Mean (s.d.)	Range	N	N (%)/Mean (s.d.)	Range
**Body Composition**									
Weight (kg)	27	64.5 ± 16	43–101.5	10	67.7 ± 19.5	44–101	18	69.3 ± 16.6	46.3–104.6
Height (cm)	27	166.1 ± 9	153–185	15	165.5 ± 8.0	155–182	19	166.7 ± 8.7	154–184.1
BMI (Kg/m2)	27	23.3 ± 5.1	16.6–33.9	10	24.7 ± 6.1	16.8–33.7	18	24.7 ± 5.0	17.4–33.8
BMI Centile	27	60.9 ± 35.2	1–99	10	66.4 ± 40.6	2–99	18	69.3 ± 34.3	6–99
BMI Category									
** **<18.5	27	6 (22.2%)	-	10	2 (20%)	-	10	2 (20%)	-
** **18.5–25	27	13 (48.2%)	-	10	3 (30%)	-	10	3 (30%)	-
** **25–30	27	4 (14.8%)	-	10	2 (20%)	-	10	2 (20%)	-
** **>=30	27	4 (14.8%)	-	10	3 (30%)	-	10	3 (30%)	-
** **Underweight <5th percentile	27	2 (7%)	-	10	2 (20%)	-	18	0 (-)	-
** **Healthy weight 5th percentile—85th percentile	27	15 (56%)	-	10	3 (30%)	-	18	9 (50%)	-
** **Overweight 85th percentile—95th percentile	27	3 (11%)	-	10	1 (10%)	-	18	3 (17%)	-
** **Obese >95th percentile	27	7 (26%)	-	10	4 (40%)	-	18	6 (33%)	-
Waist Circumference (cm)	5	86.1 ± 12.7	75–106	2	98.1 ± 8.6	-^a^	5	80.8 ± 16.1	66–100
**Heart Rate and Blood Pressure**									
Systolic Blood Pressure	27	117.1 ± 11.8	95–141	14	116.3 ± 14	90–145	16	115.6 ± 15.8	95–149
Diastolic Blood Pressure	23	75.3 ± 14.9	56–94	13	70.8 ± 17.8	45–104	16	72.8 ± 10.0	54–90
Pulse Rate (bpm)	25	85.2 ± 14.7	52–115	12	88.6 ± 14.1	68–123	15	86.7 ± 17.8	65–117
**Blood Results**									
HbA1c (mg/dL)	19	110.7 ± 16.8	96.5–173.4	6	107.1 ± 8.3	96.5–120.2	13	103.9 ± 8.3	93.9–120.2
Prolactin (mU/L)	22	226.6 ± 136.5	74–620	5	285.2 ± 95.5	164–378	11	239.7 ± 171.9	56–701
HDL (mmol/L)	21	1.2 ± 0.4	0.81–2.7	6	1.2 ± 0.3	0.9–1.5	11	1.3 ± 0.5	0.7–2.6
LDL (mmol/L)	5	2.5 ± 0.7	1.5–3.3	0	-	-	5	2.1 ± 0.6	13.-2.6
Triglycerides (mmol/L)	22	1.2 ± 0.8	0.5–3.9	6	1.6 ± 0.7	0.5–2.4	11	1.0 ± 0.4	0.4–1.8
Total Cholesterol (mmol/L)	23	4.2 ± 0.9	3–6	6	4.1 ± 1.0	3.4–6	10	4.0 ± 1.1	2.5–5.8
**Full Blood Count**									
Haemoglobin	25	143.7 ± 59.7	103–424	10	130 ± 17	100–157	12	127.1 ± 19.1	92–156
Red Blood Cell (x10^12/L)	19	4.7 ± 0.8	3.74–7.5	10	4.5 ± 0.5	3.5–5.4	12	4.5 ± 0.6	3.6–5.3
White Blood Cell	22	5.7 ± 1.7	3–9.1	11	6.5 ± 2.8	2.4–10.3	12	6.6 ± 2.3	2.8–9.8
B12	15	478.1 ± 247.9	210–1,055	1	320	-	4	289.8 ± 62.0	203–348
Vitamin D	1	66.7	-	0	-	-	-	-	-
Iron	1	10.1	-	0	-	-	0	-	-
**ECG**									
Completed recently	27	27 (100%)	-	8	8 (100%)	-	8	8 (100%)	-
**Prevalence of other conditions**									
Asthma	27	3 (11.1%)	-	25	2 (8%)	-	25	1 (4%)	-
Other^b^	27	15 (56%)	-	25	12 (67%)	-	25	16 (64%)	-
None disclosed	27	10 (37%)	-	25	10 (40%)	-	25	7 (28%)	-
**Number of non-psychotropic medications prescribed**									
0	27	12 (44.4%)		24	15 (62.5%)		25	17 (68%)	
1	27	6 (22.2%)		24	4 (16.7%)		25	4 (16%)	
2	27	3 (11.1%)		24	1 (4.2%)		25	3 (12%)	
3	27	4 (14.8%)		24	2 (8.3%)		25	0	
4+	27	2 (7.4%)		24	2 (8.3%)		25	1 (4%)	

a Not reported to maintain anonymity.

b Other includes: Nutritional and Metabolic conditions (Under/Overweight, Obese, Disordered Eating, Dyslipidaemia, Diabetes, Anaemia, Elevated Iron, Vitamin D Insufficiency, Hyperthyroidism, Haemochromatosis, Neutropenia, Celiac Disease), Gastrointestinal/Genitourinary Conditions (Constipation, Urinary Tract Infection, Double Incontinence, Rectal Bleeding with vascular abnormalities, Phimosis), Dermatological Conditions (Psoriasis, Corns, Sebaceous Cyst, Ganglion), Ear Nose Throat Conditions (Cholesteatoma), Musculoskeletal Conditions (Osgood-Schlatter Disease), Impaired Renal Functioning, Non-Epileptic Seizures.

Physical health comorbidities were observed across all timepoints, with metabolic conditions such as diabetes and dyslipidaemia (according to higher-than-normal range observed via blood tests) having been observed across timepoints, affecting 44% at baseline and 6-month follow-up. Other physical health conditions reported over time appeared to have increased (15%–28%) and encompassed a wide range of conditions including gastrointestinal, dermatological, genitourinary and musculoskeletal conditions. The proportion of people who disclosed no additional physical health conditions decreased over time—indicative of increased rates of physical health comorbidities reported over time. Similarly, more than half of all participants were prescribed medication for physical health conditions, but this declined over time.

#### Lifestyle/behavioural

Table 5 contains behavioural and lifestyle outcomes. Physical activity levels were consistently low across all timepoints. There was a slight decrease in self-reported sedentary activity at 6-months (8.4 h, compared with 9.1 h at both baseline and 3-month follow-up). Time spent walking, and engaging in sport remained low with minimal changes observed. Self-reported physical fitness according to the IFIS [Ortega et al., 2011 (20)] was average at baseline and generally increased over time. General physical fitness, cardiorespiratory fitness, muscular strength, speed/agility and flexibility all showed good to moderate increases in responses such as “good” and “very good”. Objectively measured fitness (using the 6-minute walk test) was limited due to restrictions attending ward space and is only reported for baseline. Due to low levels of completion, it was not possible to compare objectively and subjectively reported fitness.

**Table 5:** Lifestyle and behavioural outcomes.

Measure	Baseline			3-month follow-up			6-month follow-up		
	N	Mean (s.d.)	Range	N	Mean (s.d.)	Range	N	Mean (s.d.)	Range
**Physical Activity (SIMPAQ)**									
Time spent in bed at night (hours)	25	9.4 ± 1.9		18	9.1 ± 2.2		13	9.3 ± 2.6	
Sedentary activity per day (hours)	25	9.1 ± 4.4		18	9.1 ± 3.5		13	8.4 ± 3.1	
Walking per day (hours)	24	1.2 ± 1.4		17	1.8 ± 1.2		13	1.4 ± 1.2	
Sport/Exercise per day (hours)	25	0.3 ± 0.6		15	0.2 ± 0.3		13	0.3 ± 0.7	
Other activities per day (hours)	25	0.4 ± 0.7		18	0.4 ± 0.4		13	0.5 ± 0.4	
**Dietary Intake (24 h diet recall)**									
Number of Meals	25	1.8 (±1)	1–5	19	1.79 (±1.08)	0–4	13	1.85 (±0.88)	1–3
Number of Snacks	25	2.2 (±1.5)	0-6	19	2.47 (±2.09)	0–8	13	1.62 (±1.19)	0–4
Tea/Coffee (Mug)	25	1.7 (±0.8)	1-3	19	1.75 (±1.5)	1–4	13	1.00 (-)	1
Portions of Fruit	25	1.7 (±0.6)	1-2	19	1.33 (±0.58)	1–2	13	1.00 (-)	1
Portions of Veg	25	2.3 (±0.6)	2-3	19	2.00 (±1.00)	1–3	13	2.00 (-)	2
Soft Drinks (mL)	25	0 (-)	-	19	357.78 (±121.74)	150–500	13	386.00 (±361.12)	2–990
Water (mL)	25	850 (±610.9)	250–2,500	19	718.75 (±471.27)	250–1,500	13	1,218.75 (±1,583.61)	250–5,000
Energy Drink (mL)	25	-	-	19	725 (±263.00)	500–1,000	13	633.33 (±230.94)	500–900
**HONOSCA**									
Behavioural Problems	24	7.3 (±3.3)		17	6.3 (±3.2)		12	6.3 (±3.9)	
Impairment	25	3.1 (±1.7)		18	2.8 (±2.0)		15	3.3 (±2.3)	
Symptomatic	25	6.1 (±2.6)		17	4.2 (±2.8)		14	3.5 (±2.4)	
Social	23	7.6 (±3.8)		18	6.3 (±3.1)		14	6.2 (±3.0)	
Total	23	24.1 (±9.1)		17	19.5 (±8.7)		11	20.4 (±8.9)	


	**Timepoint **	**N**	**Very Good**	**Good**	**Average**	**Poor**	**Very Poor**		
**Self-reported Fitness (IFIS)**									
General Physical Fitness	Baseline	24	2 (8.3%)	3 (12.5%)	16 (66.7%)	2 (8.3%)	1 (4.2%)		
	3-Month	17	-	4 (23.5%)	10 (58.8%)	2 (11.8%)	1 (5.9%)		
	6-Month	14	2 (14.3%)	4 (28.6%)	5 (35.7%)	3 (21.4%)	-		
Cardiorespiratory Fitness	Baseline	25	4 (16%)	1 (4%)	6 (24%)	9 (36%)	5 (20%)		
	3-Month	17	1 (5.9%)	2 (11.8%)	4 (23.5%)	7 (41.2%)	3 (17.7%)		
	6-Month	14	1 (7.1%)	3 (21.4%)	4 (28.6%)	6 (42.9%)	-		
Muscular Strength	Baseline	25	2 (8%)	2 (8%)	14 (56%)	6 (24%)	1 (4%)		
	3-Month	17	3 (17.7%)	2 (11.8%)	9 (52.9%)	2 (11.8%)	1 (5.9%)		
	6-Month	14	3 (21.4%)	1 (7.1%)	8 (57.1%)	2 (14.3%)	-		
Speed/Agility	Baseline	25	4 (16%)	7 (28%)	11 (44%)	3 (12%)	0 (0%)		
	3-Month	17	2 (11.8%)	2 (11.8%)	10 (58.8%)	2 (11.8%)	1 (5.9%)		
	6-Month	14	3 (21.4%)	2 (14.3%)	7 (50%)	2 (14.3%)	-		
Flexibility	Baseline	25	5 (20%)	2 (8%)	7 (28%)	9 (36%)	2 (8%)		
	3-Month	17	1 (5.9%)	2 (11.8%)	5 (29.4%)	7 (41.2%)	2 (11.8%)		
	6-Month	14	3 (21.4%)	1 (7.1%)	3 (21.4%)	6 (42.9%)	1 (7.1%)		

	**N**	**Mean**	**± SD**	**Range**					
**Six-Minute Walk**									
Total Distance (m)	5	198.0	56.1	138–274					

^Current was defined as within the past 3 months.

None of the participants reported consumption of a balanced diet at any timepoint during the study. The average number of meals per day was 1.8 across all timepoints, with participants reporting either skipping breakfast or lunch. Fruit and vegetable consumption was low and reduced over time (1.7 to 1 portion per day). Water intake rose substantially at 6-months, and both soft-drink and energy consumption were only reported at follow-up, which is consistent with restrictions on the ward. Substance use was assessed using the WHO-ASSIST, and has previously been reported [see Carney et al., in press (18)]. The most consumed substances at baseline were alcohol (*n* = 11, 44%), tobacco (*n* = 10, 40%) and cannabis (*n* = 6, 25%), with much lower rates for other substances such as cocaine and opioids. Of those who reported smoking, e-cigarette use was high (88%), followed by consumption of cigarettes (75%) and roll-ups (63%) (see Supplementary Data for full substance use information).

#### Mental health outcomes

According to the HONSOCA participants self-reported a range of impairments, some of which were severe in domains such as education, relationships with others, education and functioning, as well as difficulties with anxiety and low mood. For example, 80% of participants reported difficulties with concentration, 75% reported self-harm, and difficulties with relationships at home was reported by over half of the sample (56%). Total HONOSCA scores decreased from 24.1 ± 9.1 upon admission to 19.5 ± 8.7 at 3-months, and 20.4 ± 8.9 at 6-months. The most notable improvement came from symptomatic scores going from 6.1 ± 2.6 to 4.2 ± 2.8 at 3-months and then 3.5 ± 2.4 at 6-months. Improvements were observed in social functioning and behavioural problems which may suggest improvements in mental health and functioning over the study period.

### Adverse events/harms

Safety was assessed through tracking incidents and adverse events. Adverse events were self-disclosed to researchers at assessment timepoints. A list of expected adverse events typical of this population was established with clinical teams and people with lived experience prior to the start of the study. Therefore, only unexpected events, reported to the research team directly were reported. There were nine adverse events reported during the study; five (55.6%) cases involved the individual presenting at A&E in distress following discharge, four (44.4%) involved a readmission to inpatient care or prolonged inpatient care, two (22.2%) involved violence towards the participant or towards others and two (22.2%) involved an attempt to take life or self-harm. Events were not mutually exclusive. No adverse events were related to participation in the study.

## Discussion

### Key findings

The Y-Health study is the first prospective longitudinal study to examine the feasibility of monitoring physical health and related lifestyle factors in young people admitted to CAMHS inpatient units in the UK. The primary contribution of this work is to suggest that comprehensive physical health assessments can be feasibly conducted during inpatient admission, with high levels of recruitment, retention and baseline data completion across multiple NHS trusts. While the study was not powered to detect statistically meaningful change over time, descriptive findings suggest a high prevalence of physical health risk factors at admission, and observed across the six-month follow-up period. A substantial proportion of participants were classified as overweight or obese at baseline, and metabolic, behavioural and lifestyle risk factors were frequently present at all assessment points. These observations are consistent with previous cross-sectional and routine data studies in CAMHS inpatient populations, which have reported high rates of cardiometabolic risk, polypharmacy, and adverse health behaviours (9, 11, 15–17).

Importantly, these findings should be interpreted cautiously. The small sample size, missing follow-up data and potential cohort effects related to the COVID-19 pandemic limit inference regarding change over time. Rather, the findings are descriptive and hypothesis generating, highlighting the complex, intersecting physical and mental health needs of this population, alongside the practical challenges of monitoring physical health longitudinally, particularly, following discharge. Together, these findings suggest a need for more integrated and sustained approaches to physical health monitoring across inpatient and community mental health services.

### Clinical implications

#### Barriers to physical health monitoring

A central aim of this study was to establish the feasibility of monitoring physical health over a six-month period following admission to CAMHS inpatient units. We successfully recruited 27 participants across three NHS mental health trusts, with a high overall retention rate of 93% at six-months, demonstrating acceptability of study procedures. However, recruitment fell short of initial expectations, largely due to pandemic-related factors including fluctuating admission patterns, ward closures, isolation requirements and changes in case-mix such as increased admissions for eating disorders (such as anorexia nervosa) and longer durations of stay, with fewer overall admissions (29, 30). As we aimed to recruit consecutive admissions, the reduced intake during the study period limited our sample size. The dynamic and unpredictable nature of CAMHS inpatient environments during the COVID-19 pandemic further complicated research delivery. Staff and researchers had to adapt rapidly to changing restrictions, which often prevented face-to-face contact and disrupted research activities (Evolving CAMHS paper, 45).

Additionally, many young people were too acutely unwell to provide informed consent at admission, which limited the inclusion of individuals with psychotic disorders or first episode psychosis. Although eligibility criteria were extended from two to six-weeks post-admission to allow for people to begin treatment and stabilise, the most severely unwell individuals were still likely under-represented. This has important implications for both research and clinical practice, as these individuals may have the greatest physical health needs and poorest long-term outcomes. Future research may benefit from more inclusive approaches, such as opt-out consent for routine clinical data, or retrospective consent procedures once capacity is regained.

Despite these challenges, follow-up was achievable; however, completion of physical health assessments declined substantially following discharge. While baseline assessments during inpatient admission were well completed (93% at six-months), access to physical health data post-discharge was limited by loss of contact with participants, fragmented care pathways and restricted access to community clinical records. Multiple attempts were made to re-engage participants via family members, caregivers, and health and social care professionals, but these were often unsuccessful. Physical health measures that were embedded within routine inpatient care (such as weight, BMI and blood pressure) were consistently feasible at baseline, whereas measures requiring additional time, equipment or specialist input (such as waist circumference, blood tests and fitness assessments) were less reliably obtained, particularly post-discharge. These findings suggest that future studies and clinical services should prioritise a core set of feasible, routinely collected physical health indicators, supplemented by additional measures where resources allow.

These findings reflect broader systemic issues reported in the literature, including uncertainty regarding responsibility for physical health monitoring during transitions between inpatient, community mental health and primary care services (14, 31). Embedding physical health oversight into discharge planning, alongside clearer commissioning and shared-care pathways, may help improve continuity of monitoring and care, particularly given the high prevalence of psychotropic medication use following inpatient admission.

Self-report measures were more consistently completed across timepoints, likely reflecting the flexible data collection options introduced during COVID-19 restrictions (such as online data capture forms, pre-paid postal returns and phone interviews), and the ability for them to be completed by a range of different people such as researcher, care team, or other individuals involved in a young person's care, without the need for specialist training. However, some physical health indicators (most notably waist circumference) were frequently omitted. This may reflect staff discomfort or lack of confidence addressing sensitive topics like weight and body image, as identified in our previous qualitative work (14). These challenges have also been reported in secure CAMHS psychiatric units where institutional barriers are observed such as time constraints, limited training and low motivation which hinder physical health promotion (16, 17). Addressing these barriers requires targeted staff training and the introduction of dedicated physical health champions within inpatient teams. A positive and health-promoting ward environment can mitigate some of these effects. Initiatives such as the “Keeping Our Staff in Mind” programme demonstrate how staff-led interventions can improve both service user and staff wellbeing and promote organisational culture change (32). Staff attitudes and ward culture play a critical role in shaping young people's engagement with physical activity and health-promoting behaviours (16–18, 33). Embedding physical health into the ethos of inpatient care requires cultural change. This includes staff training, leadership buy-in and the creation of roles such as physical health champions to drive changes. A positive, pro-active ward environment and culture, supported by peers and staff members, could help mitigate some of the physical health burden experienced by young people in inpatient care.

#### Opportunities for intervention

Although this feasibility study was not designed to evaluate intervention effects or longitudinal change, the high prevalence of physical health risk factors observed across timepoints underscores the importance of early and sustained physical health monitoring in CAMHS inpatient settings. Descriptive trends suggested that some indicators of metabolic risk and health behaviours remained sub-optimal over the study period; however, these observations should be interpreted cautiously given the limited statistical power and potential COVID-19 related cohort effects.

The proportion of participants classed as overweight or obese appeared to increase over follow-up, and metabolic conditions such as dyslipidaemia, anaemia and diabetes were observed. The prevalence in this feasibility sample may appear higher than estimates reported in general population studies; however, direct comparisons are limited. This pattern is broadly consistent with previous research indicating early cardiometabolic risk in young people receiving CAMHS inpatient care (9). (For context, national UK population data indicate that approximately one third of adolescents are classified as overweight or obese). However, direct comparisons are limited by differences in setting, sample characteristics and context of COVID-19 related restrictions, which may have influenced physical activity, diet, service use and delivery and admission patterns.

While improvements in mental health symptoms and functioning were observed, challenges such as poor concentration, self-harm and interpersonal relationships remained common. Physical activity levels and dietary intake were consistently sub-optimal, aligning with previous work describing the obesogenic nature of inpatient environments and the structural barriers to healthy behaviours in these settings (12–17). High rates of polypharmacy, including antipsychotic and antidepressant prescribing, sometimes in the absence of a formal diagnosis, further emphasises the importance of regular physical health monitoring. This is particularly important, given the national concerns regarding antipsychotic use in children and young people and their associated metabolic side effects, due to limited safety data and licensing amongst this age group (34–36). Rather there should be a “start low and go slow” approach to antipsychotic prescribing with regular monitoring of side effects (37).

Low physical activity and high levels of sedentary behaviours are well-established, modifiable risk factors for poor health outcomes (38). Inconsistencies in the measurement of health behaviours, often due to reliance on self-report, and limited standardised protocols, complicates efforts to monitor and intervene effectively. These challenges were reflected in our own data, where measures such as waist circumference and dietary intake were frequently incomplete. Additionally, we did not explicitly capture vaping behaviours in this study as vaping was significantly less prevalent in 2021 when data collection began. However, recent national surveys indicate substantial increases in vaping behaviours amongst young people, highlighting the need for future research to account for evolving health behaviour trends (39).

Inpatient admission may represent a critical window for initiating physical health assessment and multidisciplinary intervention engagement, particularly given the high rates of psychotropic medication use. However, the decline in data availability following discharge suggests that inpatient-initiated monitoring must be supported by clear pathways and shared responsibility across inpatient, community and primary care services if it is to be sustained. Future work should prioritise developmentally appropriate, youth centred and co-designed interventions, supported by organisational commitment, staff training, and ward-level cultural change, to embed physical health promotion into routine care as a core therapeutic component (14, 16, 17, 40–42).

### Future research

Future research should build on these feasibility findings by conducting larger, adequately powered longitudinal studies to examine physical health trajectories and intervention effects in CAMHS inpatient populations. Such studies should prioritise outcome measures demonstrated to be feasible in routine care and consider strategies to improve post-discharge data capture, through shared care protocols, enhanced integration with primary care services and digital data collection tools. Future research should also focus on developing and evaluating scalable interventions that address the physical health needs of young people in CAMHS inpatient settings. Improved data collection methods, such as digital tools to collect outcomes, dedicated physical health champions and better integration of research activities into clinical workflows may improve data quality allowing more definitive conclusions to be made. The use of wearable technologies may offer a promising approach to complement traditional physical health measures by enabling continuous, high-density data collection on physical activity and sleep, particularly following discharge. However, future work must carefully consider issues of acceptability, equity of access, data governance, and clinical utility when integrating digital tools into research and care pathways. Further research is also needed to better understand the role of the ward environment, culture and staff attitudes in shaping physical health practices. Measures such as the M-Back questionnaire and EssenCES (Climate Evaluation Schema) may be useful to assess barriers, attitude, confidence of staff and the overall ward environment to identify modifiable targets for intervention (43, 44). Given the complexity of difficulties faced by young people in inpatient care, co-designed, developmentally appropriate interventions involving young people with lived experience will be essential to improving both physical and mental health outcomes.

### Strengths and limitations

This study has several strengths, including its prospective longitudinal design, multi-site recruitment, and high retention rate, suggesting the acceptability of physical health research in CAMHS inpatient settings. The inclusion of a broad range of physical, behavioural and mental health measures provides valuable insight into feasibility across domains of healthcare. However, several limitations must be acknowledged. The study was not powered to detect statistically meaningful change, and findings should be interpreted descriptively. Recruitment and follow-up were impacted by COVID-19 restrictions, and the exclusion of individuals who were unable to provide informed consent may have led to the underrepresentation of those with the most severe presentations. The sample was predominantly White British, which broadly reflects the ethnic composition of the adolescent population in England, although young people from minoritised ethnic backgrounds remain under-represented in inpatient research. The sample was also predominately female and recruited largely from one NHS Trust [see baseline paper, (18)], thus limiting generalisability. Attrition and incomplete follow-up data further constrained interpretation of longitudinal patterns. Despite these limitations, the study provides important feasibility evidence and identifies key challenges and priorities for future research and service development.

## Conclusion

This study provides the first longitudinal feasibility evidence on monitoring physical health among young people in CAMHS inpatient care and identifies key challenges for future outcome-focused research. Whilst descriptive findings indicated a high prevalence of physical health risk factors across timepoints, the study was not powered to detect meaningful differences and findings should be interpreted as such. Despite high retention rates, challenges in data collection post-discharge highlighted systemic gaps in physical health monitoring and transitional care. The prevalence of metabolic conditions, polypharmacy, and adverse health behaviours underscores the clear need for integrated, developmentally appropriate models of care which address both physical and mental health. Inpatient settings offer a unique opportunity for early intervention, yet current practices remain inconsistent and under-resourced. Future efforts must prioritise scalable, co-designed interventions, improved monitoring protocols and stronger collaboration between inpatient and community services.

## Data Availability

The datasets presented in this article are not readily available because in line with the original ethics approval and review, datasets are available through any reasonable requests made to the corresponding author. Requests to access the datasets should be directed to rebekah.carney@gmmh.nhs.uk.
