# Supplementary material for: Monitoring physical health in child and adolescent mental health inpatient care: findings from the Y-Health longitudinal feasibility study
Source: Front Child Adolesc Psychiatry. 2026 Jun 17;5:1731769. doi: 10.3389/frcha.2026.1731769 (PMC13319068; doi:10.3389/frcha.2026.1731769)
Supplement: Supplementary file 1 [file Table1.docx]

# STROBE Checklist for Cohort Studies – Y-Health Feasibility Study

STROBE Statement—Checklist of items that should be included in reports of ***cohort studies***

|  | **Item No** | **Recommendation** |
| --- | --- | --- |
| **Title and abstract** | 1 | (*a*) Indicate the study’s design with a commonly used term in the title or the abstract |
|  |  | (*b*) Provide in the abstract an informative and balanced summary of what was done and what was found |
| **Introduction** | | |
| Background/rationale | 2 | Explain the scientific background and rationale for the investigation being reported |
| Objectives | 3 | State specific objectives, including any prespecified hypotheses |
| **Methods** | | |
| Study design | 4 | Present key elements of study design early in the paper |
| Setting | 5 | Describe the setting, locations, and relevant dates, including periods of recruitment, exposure, follow-up, and data collection |
| Participants | 6 | (*a*) Give the eligibility criteria, and the sources and methods of selection of participants. Describe methods of follow-up |
|  |  | (*b*) For matched studies, give matching criteria and number of exposed and unexposed |
| Variables | 7 | Clearly define all outcomes, exposures, predictors, potential confounders, and effect modifiers. Give diagnostic criteria, if applicable |
| Data sources/ measurement | 8* | For each variable of interest, give sources of data and details of methods of assessment (measurement). Describe comparability of assessment methods if there is more than one group |
| Bias | 9 | Describe any efforts to address potential sources of bias |
| Study size | 10 | Explain how the study size was arrived at |
| Quantitative variables | 11 | Explain how quantitative variables were handled in the analyses. If applicable, describe which groupings were chosen and why |
| Statistical methods | 12 | (*a*) Describe all statistical methods, including those used to control for confounding |
|  |  | (*b*) Describe any methods used to examine subgroups and interactions |
|  |  | (*c*) Explain how missing data were addressed |
|  |  | (*d*) If applicable, explain how loss to follow-up was addressed |
|  |  | (*e*) Describe any sensitivity analyses |
| **Results** | | |
| Participants | 13* | (a) Report numbers of individuals at each stage of study—eg numbers potentially eligible, examined for eligibility, confirmed eligible, included in the study, completing follow-up, and analysed |
|  |  | (b) Give reasons for non-participation at each stage |
|  |  | (c) Consider use of a flow diagram |
| Descriptive data | 14* | (a) Give characteristics of study participants (eg demographic, clinical, social) and information on exposures and potential confounders |
|  |  | (b) Indicate number of participants with missing data for each variable of interest |
|  |  | (c) Summarise follow-up time (eg, average and total amount) |
| Outcome data | 15* | Report numbers of outcome events or summary measures over time |
| Main results | 16 | (*a*) Give unadjusted estimates and, if applicable, confounder-adjusted estimates and their precision (eg, 95% confidence interval). Make clear which confounders were adjusted for and why they were included |
|  |  | (*b*) Report category boundaries when continuous variables were categorized |
|  |  | (*c*) If relevant, consider translating estimates of relative risk into absolute risk for a meaningful time period |
| Other analyses | 17 | Report other analyses done—eg analyses of subgroups and interactions, and sensitivity analyses |
| **Discussion** | | |
| Key results | 18 | Summarise key results with reference to study objectives |
| Limitations | 19 | Discuss limitations of the study, taking into account sources of potential bias or imprecision. Discuss both direction and magnitude of any potential bias |
| Interpretation | 20 | Give a cautious overall interpretation of results considering objectives, limitations, multiplicity of analyses, results from similar studies, and other relevant evidence |
| Generalisability | 21 | Discuss the generalisability (external validity) of the study results |
| **Other information** | | |
| Funding | 22 | Give the source of funding and the role of the funders for the present study and, if applicable, for the original study on which the present article is based |

*Give information separately for exposed and unexposed groups.

| **Section** | **STROBE Item** | **Response for Y-Health Study** | **Page Number** |
| --- | --- | --- | --- |
| **Title and abstract** | *Indicate the study’s design with a commonly used term in the title or the abstract* | Title and abstract clearly state this is a longitudinal feasibility study. | 1-2 |
| **Background/rationale** | *Explain the scientific background and rationale for the investigation being reported* | Background section clearly outlines the rationale for monitoring physical health in CAMHS inpatient settings and the evidence gap that this work fills. | 4 |
| **Objectives** | *State specific objectives, including any prespecified hypotheses* | Objectives are clearly stated in the 'Aims and Objectives' section at the end of the Background. | 5 |
| **Study design** | *Present key elements of study design early in the paper* | Study design is described in the 'Methods' section as a mixed-methods longitudinal cohort study. The methodology paper is referenced throughout from early on in the manuscript. | 5 |
| **Setting** | *Describe the setting, locations, and relevant dates, including periods of recruitment, exposure, follow-up, and data collection* | The setting is described in the methods. Three CAMHS inpatient units in the UK; recruitment from May 2021 to February 2022; follow-up at 3 and 6 months. | 5-6 |
| **Participants** | *Give the eligibility criteria, and the sources and methods of selection of participants. Describe methods of follow-up* | Eligibility criteria and recruitment procedures are detailed in the 'Participants' section. Recruitment methods and intake to the study have been detailed. | 6 |
| **Variables** | *Clearly define all outcomes, exposures, predictors, potential confounders, and effect modifiers. Give diagnostic criteria, if applicable* | Outcomes and variables are defined in the 'Outcomes & Assessments' section. A SPIRIT diagram is included with specific details of which assessments were conducted at each timepoint. | 7 & Table 1 |
| **Data sources/measurement** | *For each variable of interest, give sources of data and details of methods of assessment (measurement). Describe comparability of assessment methods if there is more than one group* | Measurement methods are described in detail in the 'Procedures' and 'Outcomes & Assessments' sections. | 8-9 |
| **Bias** | *Describe any efforts to address potential sources of bias* | Efforts to reduce bias include inclusive recruitment and adaptations for COVID-19 restrictions. | 7 |
| **Study size** | *Explain how the study size was arrived at* | No formal sample size calculation was required as study aimed to assess feasibility. This is reported in the data analysis section. | 10 |
| **Quantitative variables** | *Explain how quantitative variables were handled in the analyses. If applicable, describe which groupings were chosen and why* | Quantitative variables were summarized descriptively; no statistical testing between groups was conducted due to feasibility design and small numbers. | 10 |
| **Statistical methods** | *Describe all statistical methods, including those used to control for confounding* | Descriptive statistics only; no confounding control due to feasibility design. | 10 |
| **Participants** | *Report numbers of individuals at each stage of study—e.g., numbers potentially eligible, examined for eligibility, confirmed eligible, included in the study, completing follow-up, and analysed* | Participant flow is described in the 'Results' section and illustrated in Figure 1. | 10 and Figure 1 |
| **Descriptive data** | *Give characteristics of study participants (e.g., demographic, clinical, social) and information on exposures and potential confounders* | Baseline characteristics are reported in Table 3. Reference is made to the baseline characteristics and methodology paper. | Table 3 |
| **Outcome data** | *Report numbers of outcome events or summary measures over time* | Outcome data are reported in the results section and specifically Tables 2, 4 and 5. | 12, Table 2, 4-5. |
| **Main results** | *Give unadjusted estimates and, if applicable, confounder-adjusted estimates and their precision (e.g., 95% confidence interval). Make clear which confounders were adjusted for and why they were included* | Not applicable; feasibility study with descriptive analysis only. Main feasibility results are presented in the results section. | 11-16 |
| **Other analyses** | *Report other analyses done—e.g., analyses of subgroups and interactions, and sensitivity analyses* | No subgroup or sensitivity analyses conducted. | - |
| **Key results** | *Summarise key results with reference to study objectives* | Key findings are summarized in the 'Discussion' section. | 16 |
| **Limitations** | *Discuss limitations of the study, taking into account sources of potential bias or imprecision. Discuss both direction and magnitude of any potential bias* | Limitations are discussed in the 'Strengths and Limitations' section. | 21 |
| **Interpretation** | *Give a cautious overall interpretation of results considering objectives, limitations, multiplicity of analyses, results from similar studies, and other relevant evidence* | Interpretation is provided in the 'Discussion' and 'Conclusion' sections. | 17 |
| **Generalisability** | *Discuss the generalisability (external validity) of the study results* | Generalisability is discussed in the 'Strengths and Limitations' section. | 21 |
| **Funding** | *Give the source of funding and the role of the funders for the present study and, if applicable, for the original study on which the present article is based* | Funding source and role are described in the 'Funding' section. | 10 |
