# Supplementary material for: Monitoring physical health in child and adolescent mental health inpatient care: findings from the Y-Health longitudinal feasibility study
Source: Front Child Adolesc Psychiatry. 2026 Jun 17;5:1731769. doi: 10.3389/frcha.2026.1731769 (PMC13319068; doi:10.3389/frcha.2026.1731769)
Supplement: Supplementary file 2 [file Table2.docx]

## Supplementary Information

### Co-morbid diagnoses

| Depressive disorder | 11 |
| --- | --- |
| Adjustment disorder | 6 |
| Bipolar affective disorder | 1 |
| Mixed disorder of conduct and emotions | 1 |
| Autism spectrum disorder | 5 |
| Attention deficit hyperactivity disorder (ADHD) | 1 |
| Anxiety disorder | 2 |
| Mixed dissociative disorder | 1 |
| No formal diagnosis | 4 |
| Psychotic disorder | 2 |
| Overdose risk | 1 |
| Self-harm/suicidality | 3 |
| Emotionally unstable personality disorder | 2 |
| Insomnia | 1 |
| Anorexia nervosa | 1 |

### List of medications

| **Physical Health** | **Both Physical and Mental Health** | **Mental Health** |
| --- | --- | --- |
| Paracetamol | Promethazine | Fluoxetine |
| Omeprazole | Melatonin / Circadin | Sertraline |
| Adrenaline Epipen | Clonazepam | Venlafaxine |
| Fexofenadine |  | Quetiapine |
| Forceval Capsules |  | Olanzapine |
| Nicotine Inhaler |  | Risperidone |
|  |  | Aripiprazole |
|  |  | Lorazepam |
|  |  | Mediknet |

### Substance Use Information

| **Substance Use**  **(WHO ASSIST)** |  |  | **N (%)** |
| --- | --- | --- | --- |
|  | Alcohol Use (Current^) | 25 | 11 (44%) |
|  | Alcohol Use (Lifetime) | 25 | 17 (68%) |
|  | Tobacco Use (Current) | 25 | 10 (40%) |
|  | Tobacco Use (Lifetime) | 25 | 14 (56%) |
|  | Cannabis Use (Current) | 24 | 6 (25%) |
|  | Cannabis Use (Lifetime) | 25 | 11 (44%) |
|  |  |  |  |
| **Tobacco Use Questionnaire** |  |  | **N (%)** |
|  | Smokes Cigarettes | 8 | 6 (75%) |
|  | Smokes Roll-ups | 8 | 5 (62.5%) |
|  | Smokes Daily | 7 | 4 (57.1%) |
|  | Smokes Weekly (Or Less Often) | 7 | 3 (42.9%) |
|  | Quantity Smoked (Per Day): 0-5 | 7 | 4 (57.1%) |
|  | Quantity Smoked (Per Day): 6-10 | 7 | 3 (42.9%) |
|  |  |  |  |
|  | Uses E-cigarettes | 8 | 7 (87.5%) |
|  | Uses E-cigarettes Daily (Or Almost Daily) | 6 | 3 (50%) |
|  | Uses E-cigarettes Weekly (Or Less Often) | 8 | 3 (50%) |
|  | Quantity Smoked (Per Day): 6-10 | 6 | 2 (33.3%) |
|  | Quantity Smoked (Per Day): 11-15 | 6 | 2 (33.3%) |
|  | Quantity Smoked (Per Day): 15+ | 6 | 2 (33.3%) |

|  |  |  | **N** | **N (%)** |  |  |  |  |
| --- | --- | --- | --- | --- | --- | --- | --- | --- |
| **Substance Use**  **(WHO ASSIST)** |  |  |  |  |  |  |  |  |
|  | Cocaine Use (Current^) |  | 24 | 2 (8.3%) |  |  |  |  |
|  | Cocaine Use (Lifetime) |  | 25 | 2 (8%) |  |  |  |  |
|  | Amphetamine Use (Current) |  | 24 | 2 (8.3%) |  |  |  |  |
|  | Amphetamine Use (Lifetime) |  | 25 | 3 (12%) |  |  |  |  |
|  | Inhalant Use (Current) |  | 24 | 1 (4.2%) |  |  |  |  |
|  | Inhalant Use (Lifetime) |  | 25 | 2 (8%) |  |  |  |  |
|  | Sedative Use (Current) |  | 24 | 1 (4.2%) |  |  |  |  |
|  | Sedative Use (Lifetime) |  | 25 | 1 (4%) |  |  |  |  |
|  | Hallucinogen Use (Current) |  | 24 | 1 (4.2%) |  |  |  |  |
|  | Hallucinogen Use (Lifetime) |  | 25 | 2 (8%) |  |  |  |  |
|  | Opioid Use (Current) |  | 24 | 3 (12.5%) |  |  |  |  |
|  | Opioid Use (Lifetime) |  | 25 | 4 (16%) |  |  |  |  |
|  |  |  |  |  |  |  |  |  |
| **Substance Abuse**  **Low/Moderate/High**  **(WHO ASSIST)** |  |  | Baseline |  | 3-Month |  | 6-Month |  |
|  | Tobacco | Low | 24 | 16 (66.7%) | 18 | 11 (61.1%) | 18 | 11 (61.1%) |
|  |  | Moderate | 24 | 6 (25.0%) | 18 | 7 (38.9%) | 18 | 7 (38.9%) |
|  |  | High | 24 | 2 (8.3%) | 18 | 0 (-) | 18 | 0 (-) |
|  | Alcohol | Low | 24 | 19 (79.2%) | 18 | 15 (83.3%) | 18 | 15 (83.3%) |
|  |  | Moderate | 24 | 3 (12.5%) | 18 | 2 (11.1%) | 18 | 2 (11.1%) |
|  |  | High | 24 | 2 (8.3%) | 18 | 1 (5.6%) | 18 | 1 (5.6%) |
|  | Cannabis | Low | 24 | 17 (70.8%) | 18 | 12 (66.7%) | 18 | 12 (66.7%) |
|  |  | Moderate | 24 | 5 (20.8%) | 18 | 5 (27.8%) | 18 | 5 (27.8%) |
|  |  | High | 24 | 2 (8.3%) | 18 | 1 (5.6%) | 18 | 1 (5.6%) |
|  | Cocaine | Low | 24 | 22 (91.7%) | 17 | 16 (94.1%) | 17 | 16 (94.1%) |
|  |  | Moderate | 24 | 1 (4.2%) | 17 | 1 (5.9%) | 17 | 1 (5.9%) |
|  |  | High | 24 | 1 (4.2%) | 17 | 0 (-) | 17 | 0 (-) |
|  | Amphetamine-type | Low | 24 | 21 (87.5%) | 18 | 16 (88.9%) | 18 | 16 (88.9%) |
|  |  | Moderate | 24 | 3 (12.5%) | 18 | 2 (11.1%) | 18 | 2 (11.1%) |
|  |  | High | 24 | 0 (0.0%) | 18 | 0 (-) | 18 | 0 (-) |
|  | Inhalants | Low | 24 | 22 (91.7%) | 18 | 17 (94.4%) | 18 | 17 (94.4%) |
|  |  | Moderate | 24 | 2 (8.3%) | 18 | 1 (5.6%) | 18 | 1 (5.6%) |
|  |  | High | 24 | 0 (0.0%) | 18 | 0 (-) | 18 | 0 (-) |
|  | Sedatives | Low | 24 | 23 (95.8%) | 18 | 17 (94.4%) | 18 | 17 (94.4%) |
|  |  | Moderate | 24 | 1 (4.2%) | 18 | 1 (5.6%) | 18 | 1 (5.6%) |
|  |  | High | 24 | 0 (0.0%) | 18 | 0 (-) | 18 | 0 (-) |
|  | Hallucinogens | Low | 24 | 23 (95.8%) | 18 | 18 (100%) | 18 | 18 (100%) |
|  |  | Moderate | 24 | 1 (4.2%) | 18 | 0 (-) | 18 | 0 (-) |
|  |  | High | 24 | 0 (0.0%) | 18 | 0 (-) | 18 | 0 (-) |
|  | Opioids | Low | 24 | 21 (87.5%) | 18 | 15 (83.3%) | 18 | 15 (83.3%) |
|  |  | Moderate | 24 | 3 (12.5%) | 18 | 2 (11.1%) | 18 | 2 (11.1%) |
|  |  | High | 24 | 0 (0.0%) | 18 | 1 (5.6%) | 18 | 1 (5.6%) |
|  |  |  |  |  |  |  |  |  |
|  |  |  |  |  |  |  |  |  |
|  |  |  |  |  |  |  |  |  |

### Wellbeing measures

| **Measure** |  |  |  |  |  |  |  |  |
| --- | --- | --- | --- | --- | --- | --- | --- | --- |
| **HONOSCA** | | **N** | **Not at all** | **Insignificantly** | **Mild** | **Moderately** | **Severely** |  |
| Troubled by disruptive behaviour or verbal aggression | Baseline | 25 | 8 (32%) | 7 (28%) | 3 (12%) | 4 (16%) | 3 (12%) |  |
|  | 3-Month | 18 | 9 (50%) | 3 (16.7%) | 3 (16.7%) | 3 (16.7%) | - |  |
|  | 6-Month | 15 | 5 (33.3%) | 3 (20%) | 3 (20) | 4 (26.7%) | - |  |
| Lack of concentration or restlessness | Baseline | 25 | 1 (4%) | 4 (16%) | 3 (12%) | 10 (40%) | 7 (28%) |  |
|  | 3-Month | 18 | 2 (11.1%) | 0 (-) | 6 (33.3%) | 5 (27.8%) | 5 (27.8%) |  |
|  | 6-Month | 15 | 1 (6.7%) | 1 (6.7%) | 2 (13%) | 8 (53%) | 3 (20%) |  |
| Injured or harmed self on purpose | Baseline | 24 | 4 (16.7%) | 2 (8.3%) | 0 (-) | 12 (50%) | 6 (25%) |  |
|  | 3-Month | 17 | 2 (11.8%) | 2 (11.8%) | 5 (29.4%) | 5 (29.4%) | 3 (17.7%) |  |
|  | 6-Month | 13 | 4 (30.7%) | 1 (7.7%) | 3 (23%) | 3 (23%) | 2 (15%) |  |
| Problems because of alcohol, drug or solvents | Baseline | 25 | 19 (76%) | 2 (8%) | 2 (8%) | 2 (8%) | 0 (-) |  |
|  | 3-Month | 18 | 14 (77.8%) | 1 (5.6%) | 2 (11.1%) | 1 (5.6%) | 0 (-) |  |
|  | 6-Month | 15 | 11 (73%) | 2 (13%) | - | - | 2 (13%) |  |
| Difficulties keeping up with usual educational ability | Baseline | 25 | 5 (20%) | 4 (16%) | 1 (4%) | 6 (24%) | 9 (36%) |  |
|  | 3-Month | 18 | 2 (11.1%) | 4 (22.2%) | 5 (27.8%) | 2 (11.1%) | 5 (27.8%) |  |
|  | 6-Month | 15 | 3 (20%) | 1 (6.7%) | 1 (6.7%) | 5 (33.3%) | 5 (33.3%) |  |
| Physical illness or disability restricting activities | Baseline | 25 | 15 (60%) | 4 (16%) | 5 (20%) | 1 (4%) | 0 (-) |  |
|  | 3-Month | 18 | 13 (72.2%) | 1 (5.6%) | 2 (11.1%) | 2 (11.1%) | 0 (-) |  |
|  | 6-Month | 15 | 10 (66.7%) | 1 (6.7%) | 2 (13%) | 1 (6.7%) | 1 (6.7%) |  |
| Troubled by hearing voices, seeing things suspicious or abnormal | Baseline | 25 | 4 (16%) | 5 (20%) | 6 (24%) | 4 (16%) | 6 (24%) |  |
|  | 3-Month | 17 | 7 (41.2%) | 4 (23.5%) | 1 (5.9%) | 1 (5.9%) | 4 (23.5%) |  |
|  | 6-Month | 14 | 8 (57%) | 2 (14%) | 1 (7%) | 1 (7%) |  |  |
| Self-induced vomiting or head/stomach aches | Baseline | 25 | 14 (56%) | 2 (8%) | 5 (20%) | 3 (12%) | 1 (4%) |  |
|  | 3-Month | 18 | 13 (72.2%) | 1 (5.9%) | 3 (16.7%) | 1 (5.9%) | - |  |
|  | 6-Month | 15 | 11 (73%) | 2 (13%) | 1 (6.7%) | 1 (6.7%) | - |  |
| Feeling low or anxious mood or troubled by fears/obsessions | Baseline | 25 | 0 (-) | 3 (12%) | 5 (20%) | 6 (24%) | 11 (44%) |  |
|  | 3-Month | 18 | 4 (22.2%) | 2 (11.1%) | 1 (5.6%) | 6 (33.3%) | 5 (27.8%) |  |
|  | 6-Month | 14 | 2 (14%) | 3 (21%) | 3 (21%) | 4 (28%) | 2 (14%) |  |
| Lack of satisfactory friendships or bullying | Baseline | 25 | 10 (40%) | 3 (12%) | 6 (24%) | 2 (8%) | 4 (16%) |  |
|  | 3-Month | 18 | 9 (50%) | 5 (27.8%) | 1 (5.6%) | 2 (11.1%) | 1 (5.6%) |  |
|  | 6-Month | 15 | 8 (53%) | 4 (26.7%) | 2 (13%) | 1 (6.7%) | - |  |
| Found it difficult to look after self or take responsibility | Baseline | 25 | 3 (12%) | 5 (20%) | 7 (28%) | 7 (28%) | 3 (12%) |  |
|  | 3-Month | 18 | 3 (16.7% | 1 (5.6%) | 5 (27.8%) | 5 (27.8%) | 4 (22.2%) |  |
|  | 6-Month | 15 | 5 (33.3%) | 3 (20%) | 4 (26.7%) | - | 3 (20%) |  |
| Troubled by relationships in your family or substitute home | Baseline | 23 | 5 (21.7%) | 5 (21.7%) | 5 (21.7%) | 1 (4.3%) | 7 (30.4%) |  |
|  | 3-Month | 18 | 8 (44.4%) | 3 (16.7%) | 3 (16.7%) | 3 (16.7%) | 1 (5.6%) |  |
|  | 6-Month | 14 | 5 (35.7%) | 1 (7%) | 5 (35.7%) | 2 (14%) | 1 (7%) |  |
| Stopped attending education | Baseline | 25 | 5 (20%) | 4 (16%) | 5 (20%) | 7 (28%) | 4 (16%) |  |
|  | 3-Month | 18 | 5 (27.8%) | 3 (16.7%) | 3 (16.7%) | 4 (22.2%) | 3 (16.7%) |  |
|  | 6-Month | 15 | 4 (26.7%) | - | 3 (20%) | 2 (13%) | 6 (40%) |  |
|  |  |  |  |  |  |  |  |  |
| **Wellbeing (WHO-WI)** |  | **N** | **At no time** | **Some of the time** | **Less than half of the time** | **More than half of the time** | **Most of the time** | **All of the time** |
| I have felt cheerful and in good spirits | Baseline | 25 | 2 (8%) | 15 (60%) | 2 (8%) | 5 (20%) | 1 (4%) | - |
|  | 3-Month | 18 | 2 (11%) | 8 (44%) | 2 (11%) | 3 (17%) | 2 (11%) | 1 (6%) |
|  | 6-Month | 13 | - | 2 (15%) | 4 (31%) | 4 (31%) | 3 (23%) | - |
| I have felt calm and relaxed | Baseline | 25 | 6 (24%) | 7 (28%) | 6 (24%) | 5 (20%) | 1 (4%) | - |
|  | 3-Month | 18 | 1 (6%) | 8 (44%) | 5 (28%) | 1 (6%) | 3 (17%) | - |
|  | 6-Month | 15 | - | 5 (33%) | 7 (47%) | 2 (13%) | 1 (7%) | - |
| I have felt active and vigorous | Baseline | 25 | 8 (32%) | 7 (28%) | 3 (12%) | 6 (24%) | 1 (4%) | - |
|  | 3-Month | 18 | 3 (17%) | 8 (44%) | 2 (11%) | 4 (22%) | 1 (6%) | - |
|  | 6-Month | 15 | 1 (7%) | 2 (13%) | 9 (60%) | 1 (7%) | 2 (13%) | - |
| I woke up feeling fresh and rested | Baseline | 24 | 11 (45.8%) | 5 (20.8%0 | 2 (8.3%) | 5 (20.8%) | 1 (4.2%) | - |
|  | 3-Month | 18 | 7 (39%) | 6 (33%) | 2 (11%) | 2 (11%) | - | 1 (6%) |
|  | 6-Month | 15 | 4 (27%) | 5 (33%) | 3 (20%) | 2 (13%) | 1 (7%) | - |
| My daily life has been filled with things that interest me | Baseline | 25 | 6 (24%) | 6 (24%) | 6 (24%) | 3 (12%) | 4 (16%) | - |
|  | 3-Month | 18 | 2 (11%) | 7 (39%) |  | 3 (17%) | 4 (22%) | 2 (11%) |
|  | 6-Month | 15 | 1 (7%) | 9 (60%) | 2 (13%) | 1 (7%) | 2 (13%) | - |
|  |  |  |  |  |  |  |  |  |
